# ﻿Revision of the Tomoderinae (Coleoptera, Anthicidae). Part V. Three new *Macrotomoderus* Pic, 1901 from continental China and an updated key to the Palaearctic species

**DOI:** 10.3897/zookeys.1218.134413

**Published:** 2024-11-21

**Authors:** Dmitry Telnov

**Affiliations:** 1 Coleopterological Research Center, Institute of Life Sciences and Technology, Daugavpils University, Vienības iela 13, LV–5401, Daugavpils, Latvia Daugavpils University Daugavpils Latvia; 2 Department of Life Sciences, Natural History Museum, Cromwell Road, SW7 5BD, London, UK Natural History Museum London United Kingdom; 3 Institute of Biology, University of Latvia, O. Vācieša iela 4, LV-1004, Rīga, Latvia University of Latvia Rīga Latvia

**Keywords:** Ant-like flower beetles, identification, morphology, taxonomy

## Abstract

Descriptions of the following three new species of *Macrotomoderus* Pic, 1901 from continental China are provided: *M.blinsteini***sp. nov.**, *M.hirsutus***sp. nov.**, and *M.turpiculus***sp. nov.** The available identification key to the Palaearctic *Macrotomoderus* species is supplemented and updated.

## ﻿Introduction

This is the fifth work devoted entirely to the study of the species of *Macrotomoderus* Pic, 1901 and the fourth restricted to the Palaearctic species (see Telnov 1998, 2007b, 2018, 2022). *Macrotomoderus* is the second most speciose among the six genera of Tomoderinae Bonadona, 1961 (Chandler 2010; Telnov 2022; Telnov and Gusakov 2023). *Macrotomoderus* was originally erected to include a species from the Greater Sunda Islands, Sumatra (Pic 1901). Numerous species were described under the name of *Derarimus* Bonadona, 1978 which is now considered a junior synonym of *Macrotomoderus* (Bonadona 1978; Telnov 2007a). The distinctive morphological features of *Macrotomoderus* were re-defined by Telnov (2007a). The geographical distribution of *Macrotomoderus* is vast and stretches from the Indo–Australian Archipelago (the Greater Sunda Islands southwards including Java), mainland SE Asia (Uhmann 1993, 1994a, 1994b, 1994c, 1996a, 1996b, 1998, 1999; Telnov 2004, 2007a), the Philippine Archipelago (Uhmann 1994a; Telnov 2023) towards the Indian Subcontinent (Bonadona 1978), China, Japanese and Taiwan archipelagos (Telnov 2020, 2022). One hundred and forty-six species are currently attributed to *Macrotomoderus*, of which 96 are Oriental and 50 Palaearctic (author’s unpublished checklist and references herein). Additional species are described in the present paper.

The aim of the current paper is to present descriptions and illustrations of three *Macrotomoderus* species new to science from continental China and to provide an updated key to the Palaearctic species of the genus.

## ﻿Material and methods

All taxa are listed in alphabetical order (except in the key) since a phylogenetic arrangement is not yet possible. Paired morphological structures are generally treated as singular in text. For morphological studies, a Leica S6D binocular stereomicroscope (Leica Microsystems, Wetzlar, Germany) was used. Habitus images were produced with a Canon EOS 5D SLR camera (Canon Co., Tokyo, Japan) and a Canon MP-E 65 mm macro lens (Canon Co., Tokyo, Japan). Genitalia were relaxed in KOH solution, mounted on microscope slides, and fixed in dimethyl hydantoin formaldehyde (DMHF) for study and imaging; after the study, genitalia were mounted on the same slides with corresponding specimens and fixed in DMHF. Genitalia were studied and imaged using an AmScope BH 200 light microscope (AmScope Co., Los Angeles, U.S.A.) with an attached external Sony DSC–WX100 (Sony Co., Tokyo, Japan) digital camera for imaging. Helicon Focus 7 software (Helicon Soft, Kharkiv, Ukraine) was used for image stacking. Further image manipulations were done using GNU Image Manipulation Program (GIMP).

Label text is reproduced verbatim and enclosed in double quotation marks. Labels, if more than one on the same specimen, are separated by a double slash. All type specimens of the new species are provided with a black framed label on red paper with “HOLOTYPUS” or “PARATYPUS”. Author’s comments are given in square brackets.

Acronyms for scientific collections:

**DTC** Collection Dmitry Telnov, Rīga, Latvia;

**IBC** Working collection Igor Belousov, Saint Petersburg, Russia;

**NME**Naturkundemuseum Erfurt, Erfurt, Germany.

## ﻿Results

### ﻿Taxonomic account

**Class Insecta Linnaeus, 1758**

**Order Coleoptera Linnaeus, 1758**

**Suborder Polyphaga Emery, 1886**

**Superfamily Tenebrionoidea Latreille, 1802**

**Family Anthicidae Latreille, 1819**

**Subfamily Tomoderinae Bonadona, 1961**

#### ﻿Genus Macrotomoderus

Taxon classificationAnimaliaColeopteraAnthicidae

Pic, 1901

EE0E4288-3216-5AA9-A8E5-0BE52AECF329

= Derarimus Bonadona, 1978: 655, synonymy introduced by Telnov (2007a). Type species: Derarimuscarinatus Bonadona, 1978: 655 original designation.

##### Type species.

*Macrotomoderuslatipennis* Pic, 1901: 741 by monotypy.

#### ﻿ Macrotomoderus blinsteini sp. nov.

Taxon classificationAnimaliaColeopteraAnthicidae

16087767-9A01-5174-B5E6-325FEDFBA5B2

https://zoobank.org/7A41AD57-09C9-4A48-A0E5-80CE83C7B47F

Figs 1 , 2

##### Type material designated.

***Holotype*** • ♂ NME: “CHINA: Shaanxi/Sichuan Daba Shan, pass 20 km SSE Zhenping ~1750 m leg. Schülke 12.7.2001” [printed]. ***Paratype*** • 1♀ DTC: “CHINA: Shaanxi: Dabashan; 20 km SSE Zhenping 1700 m leg. Stary 26.6.2002” [printed].

##### Measurements.

Holotype, total body length 3.9 mm; head including exposed part of cranial ‘neck’ 0.8 mm long, across eyes 0.8 mm wide, pronotum 1 mm long, maximum width 0.8 mm, minimum width 0.3 mm, elytra 2.1 mm long, combined width 1.4 mm. Paratype ♀ 4 mm long.

##### Description.

**Figure 1.**
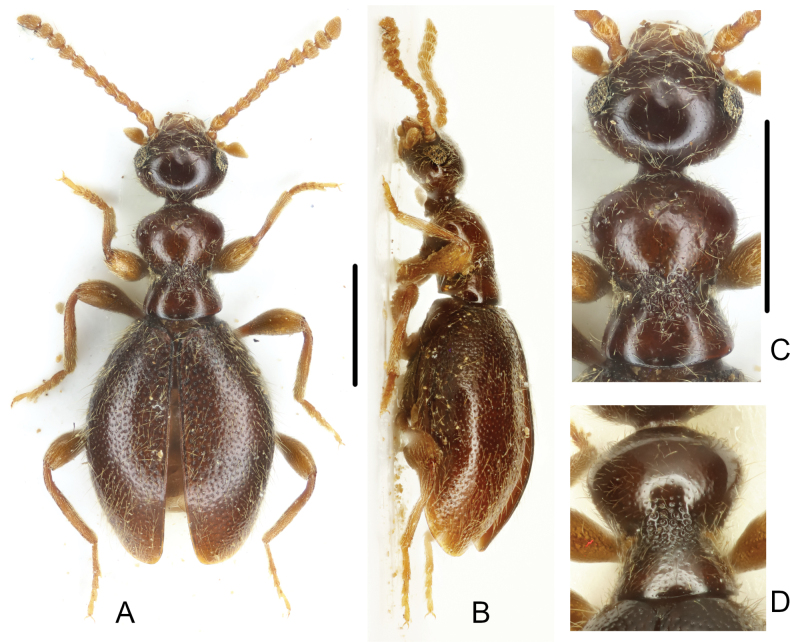
*Macrotomoderusblinsteini* sp. nov. **A** holotype ♂, habitus, dorsal view **B** ditto, lateral view **C** ditto, dorsal forebody **D** paratype ♀, pronotum, posterodorsal view. Scale bars: 1 mm.

**Figure 2.**
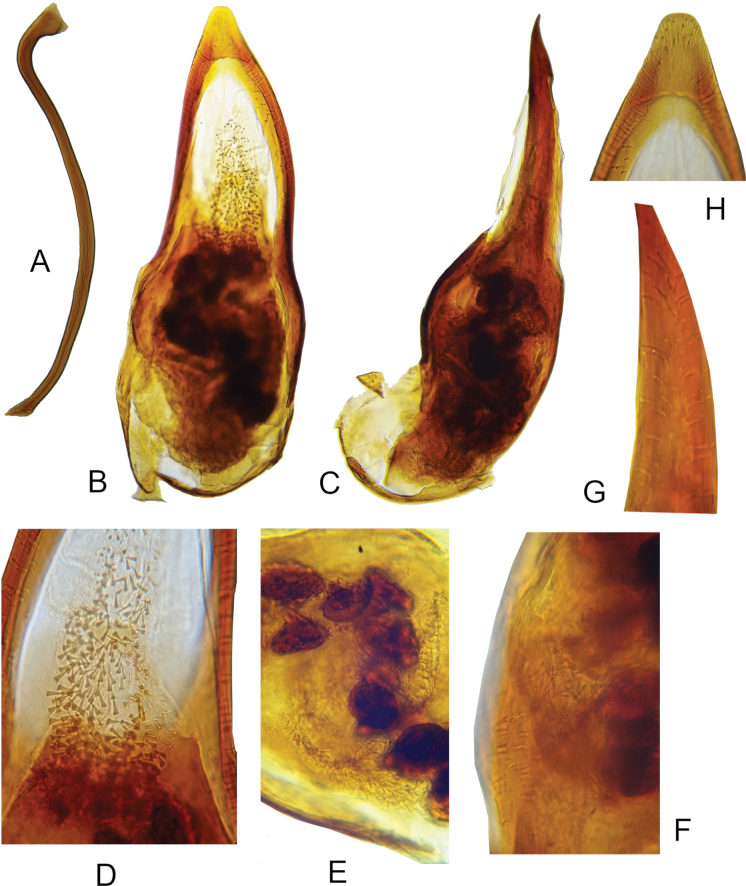
*Macrotomoderusblinsteini* sp. nov. holotype ♂, terminalia and aedeagus **A** sternite IX, lateral view **B** aedeagus, ventral view **C** ditto, lateral view **D** ditto, endophallic armature in median portion of aedeagus **E** ditto, endophallic armature in basal portion of aedeagus **F** ditto, different portion of basale **G** ditto, lateral view of apical portion with endophallic armature **H** ditto, ventral view of apex [not to scale].

Holotype, male. Dorsum and venter uniformly brown, head comparatively slightly darker. Mouthparts, antennae, palps, and legs brownish testaceous. Head transversely ovoid, glossy dorsally and ventrally, with rather small, distinctly ovoid compound eyes which are not protruding beyond lateral or dorsal outline of head. Head rounded in broad arc posterior to eyes. Head dorsal punctures minute and inconspicuous but rather deep. Intervening spaces 4–6 × as wide as diameter of punctures. Head dorsal setae inconspicuous, moderately dense, whitish to yellowish. Antenna reach base of pronotum when directed posteriad. Antennomere 3 subequal in length to antennomere 2, antennomeres 6 and 7 approximately as long as wide, 8–10 transverse, of which 9–10 strongly so. Terminal antennomere strongly asymmetrically triangular with rounded apex, ~ 1.5–1.6 × as long as penultimate antennomere. Terminal maxillary palpomere securiform. Pronotum stout, moderately glossy dorsally and laterally, narrower than head across compound eyes, with broad, medially distinctly notched (in dorsal view) postmedian lateral constriction. Front margin of anterior lobe very broadly rounded, dorsally without modifications and anterior rim. Anterior lobe barely convex in lateral view (Fig. 1B). Lateral constriction barely continues onto pronotal disc in lateral view (Fig. 1B). Lateral pronotal fovea moderately broad at lower (lateroventral) extent of laterally strongly declivous pronotal disc, somewhat widens upwards towards pronotal disc in lateral view, lateral edges of fovea carinate, narrowly separated (all in lateral view), denticle-like in dorsal view. In lateral view anterior and posterior edge of pronotal fovea covered with bristle of short golden setae except at their lower extent. Cavity in lateral wall of pronotum between lateral denticles moderate, deep. In dorsal view, lateral pronotal fovea moderately wide, anterior and posterior denticle poorly visible, glabrous. Pronotal punctures on disc similar to those on head dorsum; lateral constriction dorsally with dense, large, irregularly circular punctures with corrugate backgrounds separated by much less than puncture diameters. Dorsal pronotal setae similar to those on head dorsum, denser on lateral sides of pronotum. Few longer erect tactile setae on lateral sides of anterior lobe. Scutellar shield minute, apically rounded, glabrous, and glossy. Elytra moderately glossy, dorsally elliptical, slightly convex in lateral view, strongly widened laterally around midlength, lateral margins broadly rounded, humerus obsolete (apterous species). Elytral punctures much stronger and larger than those on dorsal forebody, more or less regularly circular, smaller, and less coarse than dorsal punctures on pronotal constriction. Punctures flatter but not much sparser on apical third of elytra. Intervening spaces ~ 1.5–2 × as wide as diameter of punctures. Elytral setae long and sparse, suberect, yellowish. Male tergite and sternite VII broadly rounded at posterior margin. Sternite IX rod-like, strongly sinuous (Fig. 2A). Aedeagus as in Fig. 2B–H, thick and bulbous in basal portion, narrowing towards subtruncate apex. Endophallic armature of numerous minute, peculiarly tack-shaped spines in apical part and, in basal portion, with large, pebble-like sclerites arranged in a kind of garland and intermixed with dense spines filling intervening spaces between sclerites (Fig. 2B–E).

***Sexual dimorphism*.** Female tergite and sternite VII broadly rounded at posterior margin, pronotum comparatively slenderer and elytra somewhat stronger constricted towards apex than in male.

##### Differential diagnosis.

This species falls in a group of species from continental China including, for example, *M.hartmanni* Telnov, 2022 and *M.korolevi* Telnov, 2022 (both from Yunnan) with the lateral constriction area of the pronotum densely and roughly but ordinary punctate and sparsely to moderately densely setose dorsally, lacking the longitudinal carinae, sulci or elongate pores. The aedeagus is differently shaped in *M.blinsteini* sp. nov., the endophallic armature is smaller, thinner and peculiarly tack-shaped, and the elytral punctures are comparatively coarser and deeper.

##### Ecology.

Collected at ~ 1700–1750 m a.s.l.

##### Distribution.

Known from the Daba Mountains in southern Shaanxi Province along boundary with Sichuan, central China.

##### Etymology.

Patronymic. This species named for Semen Blinstein (Dortmund, Germany; previously Odessa, Ukraine) – a well-known coleopterist who possesses a valuable beetle collection from southern Ukraine and is the author of some Anthicidae species from the region.

#### ﻿ Macrotomoderus hirsutus sp. nov.

Taxon classificationAnimaliaColeopteraAnthicidae

CA80F68F-D59F-5875-92FD-C5710B3F9323

https://zoobank.org/1D16E28A-F43F-40E5-88C6-82CCAB013AB1

Figs 3 , 4

##### Type material designated.

***Holotype*** • ♂ NME: “CHINA: Hubei: Dabashan; 13 km NW Muyuping 1900 m leg. Stary 16.7.2002” [printed].

##### Measurements.

Holotype, total body length 3.8 mm; head including exposed part of cranial ‘neck’ 0.9 mm long, across eyes 0.9 mm wide, pronotum 1.1 mm long, maximum width 0.9 mm, minimum width 0.35 mm, elytra 2.3 mm long, 1.7 mm combined wide.

##### Description.

**Figure 3.**
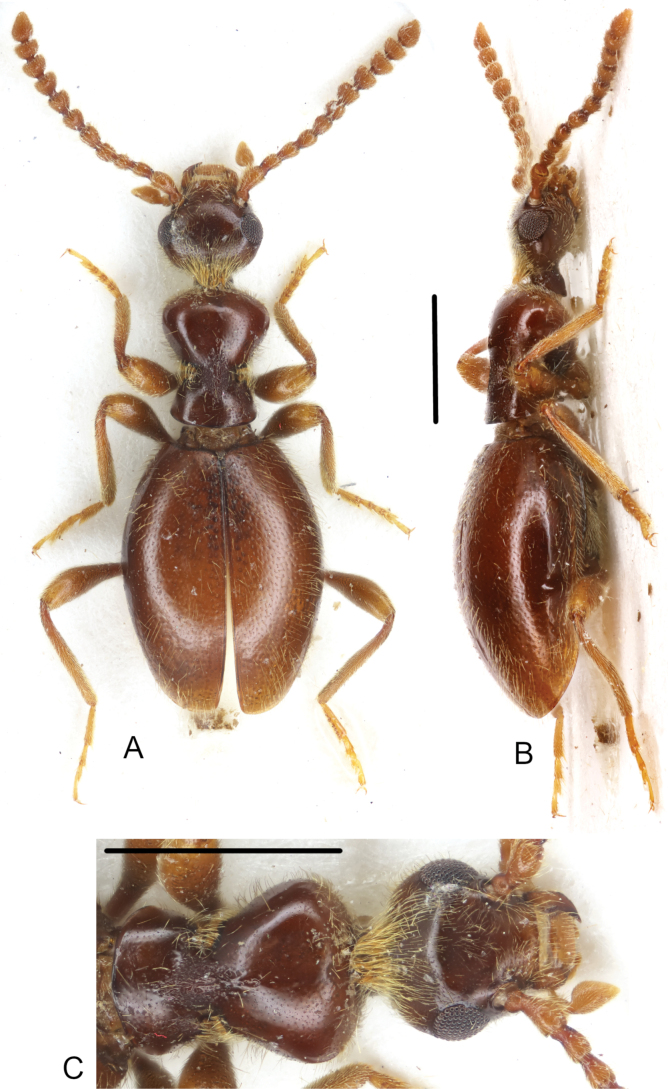
*Macrotomoderushirsutus* sp. nov. holotype ♂ **A** habitus, dorsal view **B** ditto, lateral view **C** dorsal forebody. Scale bars: 1 mm.

**Figure 4.**
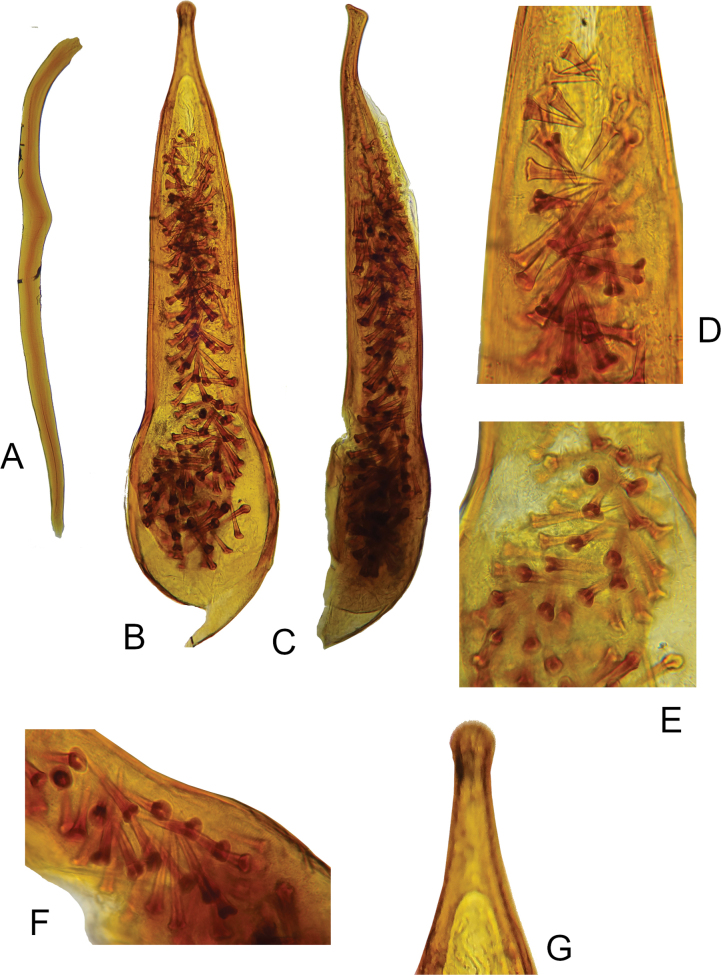
*Macrotomoderushirsutus* sp. nov. holotype ♂, terminalia and aedeagus **A** sternite IX, lateral view **B** aedeagus, ventral view **C** ditto, lateral view **D** ditto, median portion, endophallic armature **E** ditto, basal portion, endophallic armature **F** ditto, endophallic armature, lateral view of basal portion **G** ditto, ventral view of apex [not to scale].

Holotype, male. Head and pronotum brown, elytra pale brown. Mouthparts, antennae, palps, and legs pale brownish–testaceous. Head ovoid, moderately glossy dorsally and ventrally, with moderate, nearly circular compound eyes which are slightly protruding beyond lateral outline of head. Head rounded in broad arc posterior to eyes. Head dorsal punctures minute but rather deep, denser on occiput and vertex. Intervening spaces 2–4 × as wide as punctures. Head dorsal setae yellowish, inconspicuous on most of head dorsum but long and dense, forming a subconical bristle (in dorsal view) at head base and therefore concealing median part of anterior pronotal margin. Apical portions of these long head–base setae are curled and in part tangled (Fig. 3B, C). Antenna exceeds slightly beyond base of elytra when directed posteriad. Antennomere 3 subequal in length to antennomere 2, antennomeres 6–10 transverse, of which 8–10 strongly so. Terminal antennomere asymmetrically triangular with rounded apex, ~ 1.6 × as long as penultimate antennomere. Terminal maxillary palpomere securiform. Pronotum stout, moderately glossy dorsally and laterally, approximately as wide as head across compound eyes, with broad, medially strongly notched (in dorsal view) postmedian lateral constriction. Anterior lobe much wider than posterior, its front margin broadly rounded, dorsally without modifications and anterior rim. Anterior lobe nearly flat in lateral view (Fig. 3B). Lateral constriction does not continue onto disc in lateral view (Fig. 3B). Lateral pronotal fovea wide at lower (lateroventral) extent of laterally strongly declivous pronotal disc, not widens upwards towards pronotal disc in lateral view, lateral edges of fovea carinate, moderately separated and densely setose (all in lateral view), in dorsal view denticle-like. In lateral view anterior and posterior edge of pronotal fovea completely covered with bristle of short golden setae. Cavity in lateral wall of pronotum moderate. In dorsal view, lateral pronotal fovea wide, anterior and posterior denticle clearly visible, dense brush-like setose (Fig. 3A, C). Pronotal punctures on disc similar to those on head dorsum, intervening spaces 3–7 × as wide as punctures; lateral constriction dorsally with irregularly shaped and variably dense and deep, generally moderately to strongly elongated punctures; median ones particularly large and elongate, groove-like. Dorsal pronotal setae moderately dense, but much denser on lateral sides of pronotum, especially in constriction area where setation effectively conceals shape of lateral pronotal denticles. Several longer erect tactile setae on lateral sides of anterior lobe. Scutellar shield minute, apically rounded, glabrous, and glossy. Elytra moderately glossy, dorsally elongate elliptical, slightly convex in lateral view, widened laterally around midlength, lateral margins broadly rounded, humerus obsolete (apterous species). Elytral punctures stronger and larger than those on dorsal forebody, more or less regularly circular, smaller than dorsal punctures on lateral pronotal constriction. Punctures becoming more flat and somewhat sparser on apical third of elytra. Intervening spaces ~ 3–5 × as wide as diameter of punctures. Elytral setae long and sparse, suberect, yellowish. Male tergite and sternite VII broadly rounded at posterior margin. Sternite IX rod–like, slightly bisinuate (Fig. 4A). Aedeagus as in Fig. 4B–G, elongate, narrows apically, apex somewhat subspatulate, widened and rounded. Endophallic armature of numerous large peculiarly spike/nail-shaped spines (Fig. 4B–F).

***Sexual dimorphism*.** Female is unknown.

##### Differential diagnosis.

This species is readily recognized due to the presence of the dense clump of setae on male head base in the combination with the peculiar, spike/nail-like endophallic armature of the aedeagus. The shape of the aedeagus somewhat resembles that of *M.dali* Telnov, 2022 (Yunnan, China), *M.muli* Telnov, 2022 (Sichuan, China), and *M.wudu* Telnov, 2022 (Gansu, China), but all three have entirely different endophallic armatures. The head base with more or less dense setation is present in *M.conus* Telnov, 2018, *M.gracilis* Telnov, 2018, *M.microscopicus* Telnov, 2018, *M.monstrificabilis* Telnov, 2018, *M.perforatus* Telnov, 2018, and *M.schuelkei* Telnov, 2018 (all from Yunnan, China), but other morphological features and the endophallic armature are quite different.

##### Ecology.

Collected at 1900 m a.s.l.

##### Distribution.

Known from Daba Mountains in western part of Hubei Province, central China.

##### Etymology.

From Latin *hirsutus* – shaggy, hairy, bristly, referring to the bristle of setae on the head base of this species.

#### ﻿ Macrotomoderus turpiculus sp. nov.

Taxon classificationAnimaliaColeopteraAnthicidae

5077F4F1-86C2-5CAC-86CD-DB3500AD6E69

https://zoobank.org/CB509362-B8B8-47A9-9586-315EF586829B

Figs 5 , 6

##### Type material designated.

***Holotype*** • ♂ IBC: “CH, S Sichuan, S of Xichang, E slope of Mt. ´4282 ´ (NE of Dechang) 3200–2800 m, 6.05.2001 Belousov & Korolev leg.” [printed] // “Macrotomoderus sp.? n. aff. monstrificabilis [handwritten] A. Kovalev det. 20 [printed] 19” [handwritten] [label with black frame]. The holotype will be donated to a local public institution (I. Belousov, pers. comm. viii.2021). ***Paratype*** • 1♂ DTC: same labels as holotype.

##### Measurements.

Holotype, total body length 4.1 mm; head 0.8 mm long, across eyes 0.8 mm wide, pronotum 1.2 mm long, maximum width 1 mm, minimum width 0.25 mm, elytra 2.2 mm long, 1.5 mm combined wide. Paratype ♂ 4 mm long.

##### Description.

**Figure 5.**
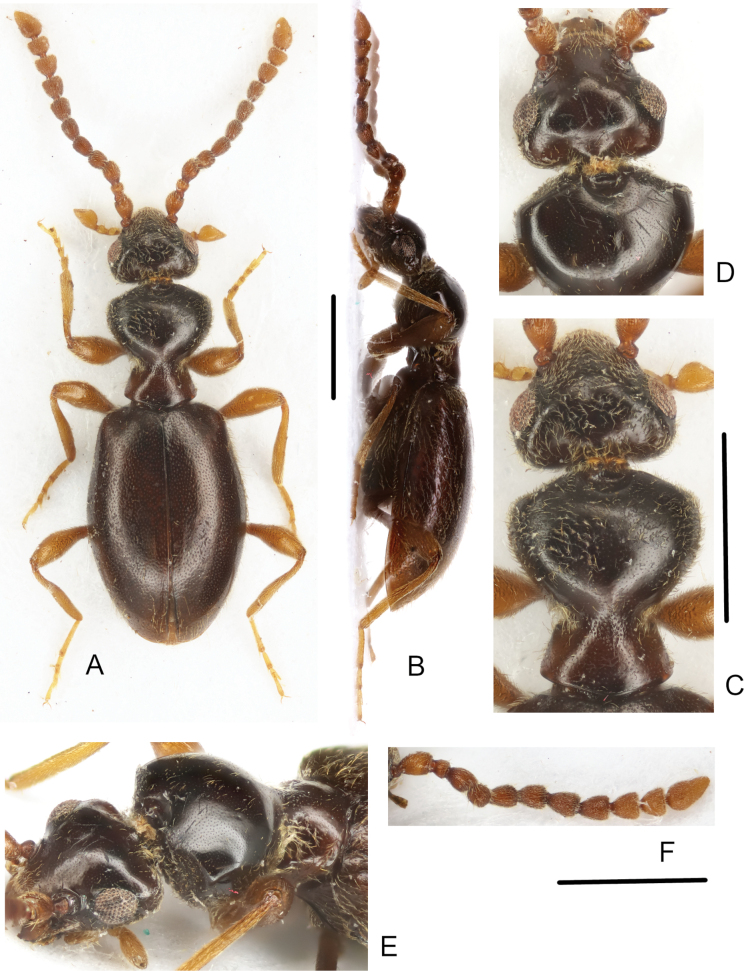
*Macrotomoderusturpiculus* sp. nov. ♂ **A** holotype, habitus, dorsal view **B** ditto, lateral view **C** ditto, dorsal forebody **D** paratype, forebody, anterodorsal view **E** ditto, latero-dorsal view **F** holotype, right antenna. Scale bars: 1 mm.

**Figure 6.**
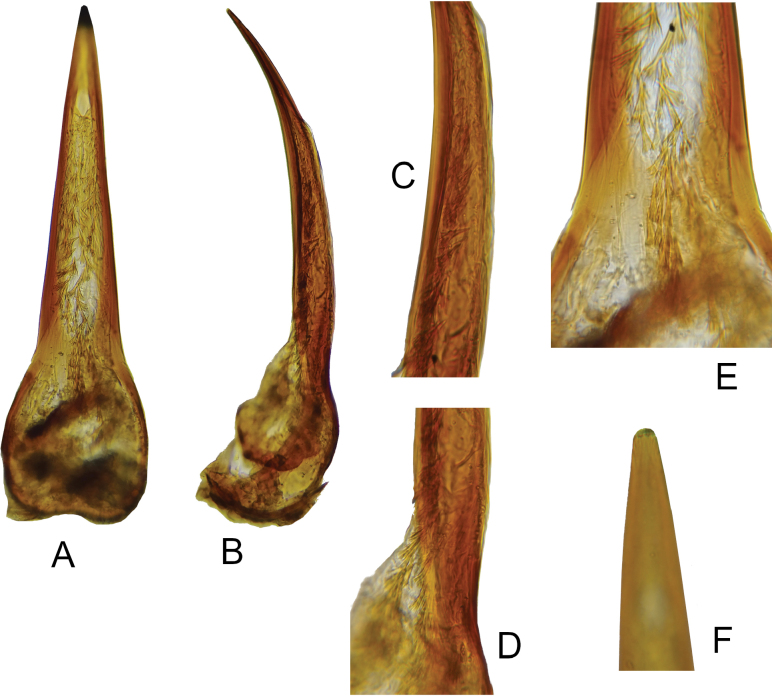
*Macrotomoderusturpiculus* sp. nov. holotype ♂, aedeagus **A** ventral view **B** lateral view **C** median portion, lateral view **D** basal portion, lateral view **E** basal portion, endophallic armature, ventral view **F** apical portion [not to scale].

Holotype, male. Dorsum and venter brown, posterior lobe of pronotum slightly paler. Mouthparts, antennae, palps, and legs brownish testaceous. Head subtriangular, moderately glossy dorsally and ventrally, with moderate, slightly ovoid compound eyes which are slightly protruding beyond lateral outline of head. Tempus short, constricted towards head base, temporal angle rounded. Head base truncate, declivous. Head dorsal punctures minute and inconspicuous, flat. Intervening spaces much wider than punctures. Head dorsal setae yellowish, short, moderately dense. Head base medially with somewhat longer, apically curled golden setae. Antenna exceeds slightly beyond base of elytra when directed posteriad. Antennomere 3 ~ 2 × as long as antennomere 2, asymmetrical, distal edge obliquely emarginate to accommodate shortened and only slightly longer than wide antennomere 4, antenna appears somewhat bent at area of antennomeres 3–5 (Fig. 5A, B, F). Antennomeres 8–10 transverse, of which antennomere 10 strongly so. Terminal antennomere strongly asymmetrically triangular with rounded apex, ~ 1.8–1.9 × as long as penultimate antennomere. Terminal maxillary palpomere securiform. Pronotum stout, moderately glossy dorsally and laterally, much wider than head across compound eyes, with moderately broad, medially strongly notched (in dorsal view) postmedian lateral constriction. Anterior lobe much wider than posterior, its front margin medially subtruncate, without anterior rim, with moderate mesal impression and transverse median ridge covered with dense golden setae which are directed antero-dorsally (Fig. 5C–E). Pronotum flattened anteroventrally each side of subtruncate median part of its anterior margin (Fig. 5D, E). Anterior lobe convex in lateral view (Fig. 5B). Lateral constriction barely continues onto disc in lateral view (Fig. 5B). Lateral pronotal fovea broad at lower (lateroventral) extent of laterally strongly declivous pronotal disc, not or barely widens upwards towards pronotal disc, lateral edges of fovea carinate, narrowly separated, nearly glabrous (all in lateral view), in dorsal view denticle-like. In lateral view anterior and posterior edge of pronotal fovea covered with a bristle of moderately long whitish setae. In dorsal view, lateral pronotal fovea wide, anterior and posterior denticle clearly visible, in part setose (Fig. 5A, C). There is an inconspicuous trace of short dorsal median longitudinal carina in lateral constriction area. Pronotal punctures on disc small and sparse, intervening spaces 5–8 × as wide as diameter of punctures; lateral constriction dorsally with irregularly ovoid and variably dense, deep punctures. Intervening spaces among punctures in laterally constricted area vary from much narrower than to twice as wide as punctures. Dorsal pronotal setae short and dense, much longer and denser at lateral pronotal fovea, in part concealing its structure in dorsal view. Few longer erect tactile setae on lateral sides of anterior lobe. Scutellar shield minute, apically rounded, moderately glossy. Elytra moderately glossy, dorsally elongate elliptical, moderately convex in lateral view, widened laterally around midlength, lateral margins broadly rounded, humerus broadly rounded. Metathoracic wings fully developed (functional). Elytral punctures stronger and larger than those on dorsal forebody, nearly as large as those on lateral constriction area, more or less regularly circular. Punctures becoming more flat and somewhat sparser on apical third of elytra. Intervening spaces ~ 2.5–4 × as wide as diameter of punctures. Elytral setae long and moderately dense, suberect, yellowish. Male tergite and sternite VII broadly rounded at posterior margin. Aedeagus as in Fig. 6, elongate and narrow, narrows and acutely angulate apically. Endophallic armature of numerous basally multifurcate spines (Fig. 6A–E).

***Sexual dimorphism*.** Female is unknown.

##### Differential diagnosis.

This species falls into a group of species from mainland China with strongly widened, apically subtruncate anterior margin of pronotum, long heavy antennae, and slender, dagger-shaped aedeagus: *M.imitator* Telnov, 2022, *M.monstratus* Telnov, 2018, and *M.monstrificabilis* Telnov, 2018 (all from Yunnan). *Macrotomoderusturpiculus* sp. nov. is peculiar due to the absence of the large elongate median projection on head base (large projection present in *M.monstrificabilis*, only small projection in *M.turpiculus* sp. nov.; cf. Fig. 5A, C, E), the truncate and declivous head base (the head base broadly rounded in the similar species, not declivous), the differently modified anterior pronotal margin (see the description) especially the anteroventrally flattened pronotum, the asymmetrical antennomere 3 and the shortened antennomere 4 (both not modified in *M.monstratus*), the wide head (the head is narrower than the pronotum in *M.imitator*), and the peculiar endophallic armature not like in other congeners.

##### Ecology.

Collected between 2800–3200 m a.s.l.

##### Distribution.

Known only from southern part of Sichuan Province, southwestern China.

##### Etymology.

From Latin *turpiculus* – misshaped, ugly, referring to the unusual body shape of this species.

### ﻿Updated key to the Palaearctic species of *Macrotomoderus*

Female features alone are generally insufficient for species delimitation; therefore, the present key is mainly based on male features. For most confident identification, the original descriptions of each species must be consulted. The present key is an update to Telnov (2022) and covers all *Macrotomoderus* taxa from the zoogeographic area as defined in the latest edition on the Palaearctic catalogue for tenebrionoid beetles (Iwan and Löbl 2020).

**Table:** 

1	Head base distinctly constricted, tapered	***M.conus* Telnov, 2018**
–	Head rounded, subtruncate, truncate or emarginate (concave) posterior to compound eyes (species with median conical projection on generally rounded to subtruncate head base should be included here)	**2**
2	Male metafemur with conspicuous, large, apically acutely pointed spine at posterior margin	***M.femoridens* Telnov, 2022**
–	Posterior margin of metafemur in both sexes without modifications	**3**
3	Head base in male with median projection, small or distinct, sometimes concealed beneath dense setae and difficult to observe	**4**
–	Head base in male without median projection	**7**
4	Head base in male truncate, head base declivous; anterior margin of pronotum with shallow median emargination and mesally with transverse ridge covered with dense antero-dorsally pointed setae; anteroventral part of pronotum flattened each side of median area (Fig. 6); endophallic armature of numerous basally multifurcate spines	***M.turpiculus* sp. nov.**
–	Head base rounded, not declivous; anterior margin of pronotum without modifications or modification is different (e.g., laterally angulate median impression with transverse ridge or simple shallow median impression), not flattened anteroventrally; endophallic armature not as above	**5**
5	Anterior margin of male pronotum with broad mesal emargination facing median part of head base, anterolateral margins of emarginated area moderately raised in dorsal aspect, appear angulate; anterior edge of pronotum in front of emargination in male forms median transverse ridge that is covered with conspicuous, in part curved, anterodorsally pointed setae	***M.lapidarius* Telnov, 2022**
–	Anterior margin of pronotum, if impressed, with margins of impression not angular	**6**
6	Anterior margin of male pronotum truncate, with shallow mesal impression; tapered projection of head base large; head with distinct tempora, compound eye twice as long as tempus; head base subtruncate	***M.monstrificabilis* Telnov, 2018**
–	Anterior margin of male pronotum broadly rounded, not impressed; projection of head base less conspicuous, smaller; head rounded in broad arc posterior to eyes, tempora not delimited	***M* . *mirabilis* Telnov, 2018**
7	Head base in male truncate when observed strictly from above, temporal angle present, obtuse; anterior margin of male pronotum with broad mesal impression facing median part of head base, anterolateral margins of impressed area slightly raised in dorsal aspect, appearing obtuse angular; anterior edge of male pronotum in front of anterior impression forms thin, low, transverse median ridge covered with conspicuous, in part curved, anterodorsally pointed setae	***M.truncatulus* Telnov, 2022**
–	Combination of features different; male head base rounded, subtruncate, or truncate but temporal angle never appearing angular	**8**
8	Total body length ≤ 2 mm	**9**
–	Total body length ≥ 2.5 mm	**10**
9	Species from Okinawa, Ryukyu Islands; anterior lobe of pronotum dorsally with inconspicuous median longitudinal carina; anterior margin of male pronotum without modifications, rounded	***M.satoi* (M. Saitô, 2003)**
–	Species from Yunnan, continental China; pronotum dorsally not carinate; anterior margin of male pronotum with mesal impression that holds small frontal projection	***M.microscopicus* Telnov, 2018**
10	Anterior margin of male pronotum with modifications – mesally impressed or projected anteriorly or provided with conspicuous, grouped, dense setae	**11**
–	Anterior margin of male pronotum evenly rounded, subtruncate or truncate, without modifications or group(s) of conspicuous setae, at most slightly impressed mesally	**28**
11	Anterior lobe of pronotum distinctly wider than head across eyes	**12**
–	Anterior lobe of pronotum nearly as wide as or narrower than head across eyes	**16**
12	Head base truncate; compound eyes conspicuously large and laterally convex; tempus distinct, much shorter than dorsal eye length	***M.boops* Telnov, 2022**
–	Head rounded in broad arc posterior to comparatively small, more or less strongly flattened compound eyes; tempus not delimited (rounded) but not much shorter than dorsal eye length	**13**
13	Male occiput not declivous or impressed posterodorsally (above insertion of cranial ‘neck’); anterior margin of male pronotum slightly impressed both sides of median projection; male antenna comparatively shorter, not exceeding median third of elytra	***M.hengduan* Telnov, 2022**
–	Male occiput slightly declivous posterodorsally or shallowly impressed posterodorsally above insertion of cranial ‘neck’; male antenna extending or nearly extending beyond median third of elytra	**14**
14	Male occiput slightly declivous posterodorsally; anterior margin of pronotum without median projection	***M.dali* Telnov, 2022**
–	Male occiput shallowly impressed posterodorsally above insertion of cranial ‘neck’; anterior margin of pronotum with distinct, median triangular projection	**15**
15	Anterior margin of pronotum truncate; lateral constriction area of pronotum dorsally with inconspicuous, short, median longitudinal carina, densely and roughly punctured both sides of it, intervening spaces much smaller than punctures	***M.imitator* Telnov, 2022**
–	Anterior margin of pronotum subtruncate; lateral constriction area of pronotum dorsally not carinate, with rather large and sparse punctures and wide, glossy, and glabrous intervening spaces	***M.monstratus* Telnov, 2018**
16	Lateral foveae of pronotum not or marginally visible in dorsal view, not forming deep notches in pronotal constriction in dorsal aspect; denticles of lateral foveae of pronotum not or only partially visible in dorsal view	**17**
–	Lateral foveae of pronotum clearly visible in dorsal view in a form of variably deep notches in sides of pronotum at constriction area; denticles of lateral foveae of pronotum generally well visible in dorsal view	**20**
17	Lateral constriction of pronotum dorsally with more or less prominent median longitudinal carina	***M.chingpo* Telnov, 2018**
–	Lateral constriction of pronotum dorsally not carinate	**18**
18.	Anterior margin of male pronotum without modifications, broadly rounded, with a group of C-like shaped (curled posteriad) posteriad-pointed setae; head base without bunch of setae	***M.angelinii* Telnov, 2022**
–	Anterior margin of pronotum in male medially elevated and projecting anteriad; head base with or without group of longer setae	**19**
19	Anterior projection of pronotum with a group of ך-like shaped (bent anteriad) anteriad-pointed setae; head base medially with a bunch of dense setae; pronotum slender, elongate, narrower than head across eyes	***M.gracilis* Telnov, 2018**
–	Anterior margin of pronotum without bent or curved setae; pronotum rather broad, approx. the width of head across eyes	***M.kawa* Telnov, 2018**
20	Dorsum of anterior lobe of pronotum or its lateral constriction area or both medially longitudinally carinate	**21**
–	Pronotum not carinate dorsally	**25**
21	Anterior lobe of pronotum dorsally with median longitudinal carina (almost complete but not touching its anterior margin or restricted to posterior half of anterior lobe), projecting or not on lateral constriction area	**22**
–	Pronotum only carinate on lateral constriction area; anterior lobe of pronotum without dorsal carina	**24**
22	Anterior margin of male pronotum not excavated, mesally with group of dense, strongly Ɔ-like shaped (curled anteriad) anteriad-pointed setae	***M.wudu* Telnov, 2022**
–	Anterior margin of male pronotum excavated, without median group of dense, curved setae	**23**
23	Aedeagus with strongly widened, bulbous basal portion, apically narrowly rounded to subacute in dorsal and ventral view; apex of aedeagus straight in lateral view	***M.transitans* Telnov, 2022**
–	Aedeagus with moderately wide, non-bulbous basale, apically clearly rounded in dorsal and ventral view; apex of aedeagus slightly curved in lateral view	***M.bordonii* Telnov, 2022**
24	Lateral constriction area of pronotum tricarinate dorsally, of which both lateral carinae less prominent and shorter than median carina; lateral pronotal foveae in dorsal view comparatively shallower and less broadly notched; dorsal setae on pronotum moderately long	***M.yunnanus* (Telnov, 1998)**
–	Lateral constriction area of pronotum dorsally unicarinate along midline; lateral pronotal foveae in dorsal view deeply and broadly notched; dorsal setae on pronotum longer	***M.perforatus* Telnov, 2018**
25	Anterior margin of male pronotum without modifications, broadly rounded, medially with a bunch of rather short and dense, apically C-shaped (curved) posteriad-pointed setae	***M.tenuis* Telnov, 2022**
–	Anterior margin of male pronotum with median emargination or projection	**26**
26	Anterior margin of male pronotum truncate, with small and shallow median emargination; anterior transverse ridge in this impression with a group of ך-like shaped (bent anteriad), antero-dorsally pointed setae raised from one pore	***M.schuelkei* Telnov, 2018**
–	Anterior margin of male pronotum rounded to broadly rounded, of different structure	**27**
27	Anterior margin of male pronotum with paired bunch of long, strongly Ɔ-like shaped (curled anteriad) anteriad-pointed setae touching cranial ‘neck’ and head base; occiput slightly declivous posterodorsally; head base broadly rounded	***M.bicrispus* Telnov, 2022**
–	Anterior margin of male pronotum mesally emarginated; anterior margin of this cavity laterally with some long, apically curved, erect setae which are meeting apically in П-like shaped arc over anterior wall of pronotum	***M.similis* Telnov, 2022**
28	Lateral constriction of pronotum dorsally with two rather large, elongate ovoid notch-like pores and 2 or 3 obtuse, transverse sulci	***M.negator* Telnov, 2007**
–	Dorsal sculpture of lateral pronotal constriction different	**29**
29	Head base with bristle of long posteriad-directed setae of subconical appearance; aedeagus as in Fig. 4, endophallic armature of peculiar, spike-nail-shaped spines	***M.hirsutus* sp. nov.**
–	Head base without subconical-like bristle of long posteriad-directed setae; shape of aedeagus and endophallic armature different	**30**
30	Setae conspicuously dense in pronotal constriction area, in dorsal view effectively concealing structure of constriction, its lateral notches, and its denticles	**31**
–	Setae more or less sparse in pronotal constriction, its structure, notches, and denticles clearly visible through setae in dorsal view	**32**
31	Aedeagus comparatively stronger elongate, basale not bulbous, apical portion strongly sinuous in lateral view; tempus rather long, slightly constricted posteriad, temporal angle broadly rounded; distribution – Sichuan Province, China	***M.muli* Telnov, 2022**
–	Aedeagus shorter and stouter, basale strongly bulbous, apical portion slightly sinuous in lateral view; head posterior to compound eyes evenly broadly rounded in arc; distribution – Zhejiang Province, China	***M.makarovi* Telnov, 2018**
32	Lateral constriction of pronotum dorsally more or less distinctly medially longitudinally carinate (sometimes only visible by sufficient light!); median carina projected or not to anterior lobe	**33**
–	Lateral constriction of pronotum dorsally more or less distinctly punctate or rugulose, not carinate	**39**
33	Dorsal median longitudinal carina of pronotal constriction projected to anterior lobe of pronotum for approx. half-length of lobe	**34**
–	Dorsal median longitudinal carina of pronotal constriction restricted to lateral constriction area or (at maximum) also its anterior and posterior slope	**36**
34	Basal half of elytra strongly punctate; pronotum paler than dark brown head and elytra; basal portion of aedeagus bulbous in lateral view	***M.andibani* Telnov, 2007**
–	Basal half of elytra comparatively less strongly punctate; dorsal body uniformly brown; aedeagus basally bulbous or not	**35**
35	Male antennomeres 9 and 10 less strongly transverse (cf. Telnov 2022: fig. 3D); anterior lobe of pronotum convex in lateral view; clypeus emarginate anterodorsally; elytra comparatively longer, elytron apically rounded	***M.belousovi* Telnov, 2022**
–	Male antennomeres 9 and 10 strongly transverse (cf. Telnov 2022: fig. 24D); anterior lobe of pronotum flattened in lateral view; clypeus truncate anterodorsally; elytra comparatively shorter, elytron apically subtruncate	***M.kabaki* Telnov, 2022**
36	Terminal antennomere broadly subtriangular, apically rounded; head darker than rest of body; pronotum comparatively slender; aedeagus apically unevenly rounded in dorsal and ventral view (Telnov 2018: fig. 100)	***M.bukejsi* Telnov, 2018**
–	Terminal antennomere elongate triangular, apically pointed; head not darker than rest of body; pronotum comparatively less slender; aedeagus apex different in dorsal and ventral view	**37**
37	At least elytra dark brown; sternite IX distinctly sinuous; in dorsal view; lateral constriction area of pronotum with dense, rough punctures continue to sides (slopes) of lateral fovea; aedeagus thick, basal portion bulbous, endophallic armature without pebble-like sclerites	***M.spurisi* Telnov, 2018**
–	Dorsum uniformly pale brown, elytra not darker than rest of body; sternite IX arched or slightly sinuous; in dorsal view sides (slopes) of lateral fovea of lateral constriction area of pronotum smooth, not densely punctured; aedeagus if thick with bulbous bosal portion than also pebble-like sclerites present in endophallic armature	**38**
38	Basale of aedeagus strongly bulbous; anterior margin of pronotum broadly rounded; lateral pronotal fovea dorsally deep and broad, lateral denticles prominent, well visible dorsally	***M.jiuhuanus* Telnov, 2007**
–	Basale of aedeagus slightly bulbous, aedeagus distinctly slenderer; anterior margin of pronotum subtruncate; lateral pronotal fovea dorsally rather narrow, lateral denticles less conspicuous, not prominent, poorly visible in dorsal view	***M.periclitatus* Telnov, 2018**
39	Lateral fovea of pronotal constriction in dorsal view on each edge with two pairs of lateral denticles (anterior and posterior, upper and lower)	**40**
–	Lateral fovea of pronotal constriction in dorsal view on each edge with one pair of lateral denticles (anterior and posterior)	**41**
40	Upper posterior denticle of lateral fovea of pronotal constriction with a brush of short, dense setae (in lateral and dorsal view); lateral constriction continues onto pronotal disc, rather deep in lateral view; compound eye moderately large	***M.sichuanus* (Telnov, 1998)**
–	No brush of setae on denticles in lateral fovea; lateral constriction vaguely continues onto disc, flat in lateral view; compound eye small	***M.darrenmanni* Telnov, 2018**
41	Species from Japanese Archipelago (Honshu, Shikoku)	***M.clavipes* (Champion, 1890)**
–	Species from Taiwan	***M.nigripennis* (Uhmann, 1994)**
–	Species from mainland China	**42**
42	Denticles of lateral pronotal fovea not visible in dorsal view; basal portion of endophallic armature of two subparallel rows of dense spines	***M.daxiangling* Telnov, 2022**
–	Denticles of lateral pronotal fovea clearly visible in dorsal view; endophallic armature different	**43**
43	Transition of lateral constriction fovea to pronotal disc is gradual; transverse section across constriction area is arc-shaped	**45**
–	Transition of lateral constriction fovea to the disc of pronotum is delimited by dorso–lateral expansion of pronotal disc; transverse section across constriction area is similar to T-shape	**44**
44	Terminal antennomere elongate; pronotal constriction area comparatively wider (in dorsal view); elytra comparatively shorter; aedeagus without apical projection, apex pointed	***M.wuliangshan* Telnov, 2018**
–	Terminal antennomere small; pronotal constriction area comparatively narrower (dorsal view); elytra comparatively more elongate; aedeagus with step-like apical projection, apex subtruncate	***M.silvicolus* Telnov, 2018**
45	At least one denticle of lateral pronotal fovea (anterior or posterior, as visible in dorsal view) with brush of very dense, short setae	**46**
–	Denticles of lateral pronotal fovea glabrous or with sparse, long setae	**47**
46	Species from Hubei, China; anterior pronotal lobe dorsally minutely punctured; lateral pronotal fovea in dorsal view rather short and narrow; lateral pronotal constriction comparatively less deep (Telnov 2018: fig. 40)	***M.kurbatovi* (Telnov, 1998)**
–	Species from Guangdong, China; anterior pronotal lobe dorsally with sparse but large and deep punctures; lateral pronotal fovea in dorsal view broad and deep; lateral pronotal constriction deeper and broader (Telnov 2022: fig. 16)	***M.hajeki* Telnov, 2022**
47	Lateral constriction area of pronotum dorsally smooth and glossy, none or only tracks of sculpture present	***M.uhmanni* (Telnov, 1998)**
–	Lateral constriction area of pronotum dorsally distinctly punctured, glossy or subopaque	**48**
48	Anterior margin of pronotum medially truncate, slightly declivous anteriorly; aedeagus stout and bulbous, strongly bi-gibbose in lateral view	***M.palaung* Telnov, 2022**
–	Anterior margin of pronotum broadly rounded to subtruncate, not declivous anteriorly; aedeagus different in lateral view	**49**
49	Dorsal body dark brown; endophallic armature garland-like (Telnov 2022: fig. 19)	***M.hartmanni* Telnov, 2022**
–	Dorsal body dark or pale brown; endophallic armature distinctly different	**50**
50	Lateral constriction area of pronotum dorsally with large, elongate, median longitudinal notch; dorsal punctures on lateral constriction area of pronotum comparatively smaller and less rough; aedeagus strongly constricted towards narrowly rounded apex (Telnov 2022: fig. 43)	***M.usitatus* Telnov, 2022**
–	Lateral constriction area of pronotum dorsally without median longitudinal notch, punctate; dorsal punctures on lateral constriction area of pronotum comparatively larger and rougher; aedeagus not strongly constricted towards apex	**51**
51	Endophallic armature of apical portion of aedeagus small, peculiarly tack-shaped (Fig. 2B, D); elytral punctures comparatively coarser and deeper	***M.blinsteini* sp. nov.**
–	Endophallic armature of apical portion of aedeagus not tack-shaped; punctures on posterior half of elytron comparatively less dense and flatter	***M.korolevi* Telnov, 2022**

## Supplementary Material

XML Treatment for Macrotomoderus

XML Treatment for Macrotomoderus blinsteini

XML Treatment for Macrotomoderus hirsutus

XML Treatment for Macrotomoderus turpiculus

## ﻿Additional information

### Author ORCIDs

Dmitry Telnov https://orcid.org/0000-0003-3412-0089

### Data availability

All of the data that support the findings of this study are available in the main text.
